# Globospiramine from *Voacanga globosa* Exerts Robust Cytotoxic and Antiproliferative Activities on Cancer Cells by Inducing Caspase-Dependent Apoptosis in A549 Cells and Inhibiting MAPK14 (p38α): In Vitro and Computational Investigations

**DOI:** 10.3390/cells13090772

**Published:** 2024-04-30

**Authors:** Joe Anthony H. Manzano, Elian Angelo Abellanosa, Jose Paolo Aguilar, Simone Brogi, Chia-Hung Yen, Allan Patrick G. Macabeo, Nicanor Austriaco

**Affiliations:** 1 The Graduate School, University of Santo Tomas, España Blvd., Manila 1015, Philippines; joeanthony.manzano.gs@ust.edu.ph; 2 UST Laboratories for Vaccine Science, Molecular Biology and Biotechnology, Research Center for the Natural and Applied Sciences, University of Santo Tomas, España Blvd., Manila 1015, Philippines; jpaguilar@ust.edu.ph; 3 Laboratory for Organic Reactivity, Discovery, and Synthesis (LORDS), Research Center for the Natural and Applied Sciences, University of Santo Tomas, España Blvd., Manila 1015, Philippines; elianangelo.abellanosa.pharma@ust.edu.ph; 4 Department of Pharmacy, University of Pisa, Via Bonanno 6, 56126 Pisa, Italy; simone.brogi@unipi.it; 5 Graduate Institute of Natural Products, College of Pharmacy, Kaohsiung Medical University, Kaohsiung 80708, Taiwan; chyen@kmu.edu.tw; 6 Department of Chemistry, College of Science, University of Santo Tomas, España Blvd., Manila 1015, Philippines; 7 Department of Biological Sciences, College of Science, University of Santo Tomas, España Blvd., Manila 1015, Philippines

**Keywords:** *Voacanga globosa*, globospiramine, spirobisindole alkaloid, cytotoxicity, antiproliferative, apoptosis, cancer, molecular docking, network pharmacology

## Abstract

Bisindole alkaloids are a source of inspiration for the design and discovery of new-generation anticancer agents. In this study, we investigated the cytotoxic and antiproliferative activities of three spirobisindole alkaloids from the traditional anticancer Philippine medicinal plant *Voacanga globosa*, along with their mechanisms of action. Thus, the alkaloids globospiramine (**1**), deoxyvobtusine (**2**), and vobtusine lactone (**3**) showed in vitro cytotoxicity and antiproliferative activities against the tested cell lines (L929, KB3.1, A431, MCF-7, A549, PC-3, and SKOV-3) using MTT and CellTiter-Blue assays. Globospiramine (**1**) was also screened against a panel of breast cancer cell lines using the sulforhodamine B (SRB) assay and showed moderate cytotoxicity. It also promoted the activation of apoptotic effector caspases 3 and 7 using Caspase–Glo 3/7 and CellEvent-3/7 apoptosis assays. Increased expressions of cleaved caspase 3 and PARP in A549 cells treated with **1** were also observed. Apoptotic activity was also confirmed when globospiramine (**1**) failed to promote the rapid loss of membrane integrity according to the HeLa cell membrane permeability assay. Network pharmacology analysis, molecular docking, and molecular dynamics simulations identified MAPK14 (p38α), a pharmacological target leading to cancer cell apoptosis, as a putative target. Low toxicity risks and favorable drug-likeness were also predicted for **1**. Overall, our study demonstrated the anticancer potentials and apoptotic mechanisms of globospiramine (**1**), validating the traditional medicinal use of *Voacanga globosa*.

## 1. Introduction

A continuous search for anticancer agents is needed given the widespread occurrence of chemotherapeutic resistance [1,2]. Among the strategies considered is targeting the apoptotic pathway [3]. Apoptosis, or the cell’s natural death, is considered a crucial mechanism to prevent cancer development [4]. However, among the hallmarks of cancer cells is their ability to evade and/or downregulate apoptotic control mechanisms and machinery [5,6]. Dysregulated apoptosis allows malignant cells to persist for a longer period, propelling mutations to accumulate and abnormal phenotypes capable of aberrant cell proliferation, increased invasion, and the disruption of differentiation mechanisms to emerge [4]. Among the known apoptosis-evasion mechanisms of cancer cells include the inhibition of caspases, disabling of apoptosis trigger signals, and upregulation of anti-apoptotic proteins such as BCL-2. Given that apoptosis evasion is a general hallmark across cancer types, targeting the cell’s own mechanism of death has been recognized as the most successful non-surgical treatment modality to eradicate cancer. Numerous anticancer agents rely on their pro-apoptotic activities to elicit their anticancer properties by either upregulating pro-apoptotic proteins or inhibiting anti-apoptotic molecules [3,7,8].

Natural products (NPs) are small molecules derived from natural origins tapped for drug discovery research owing to their biological activities and low toxicity risks [9]. In addition, they are established pharmacophore templates and drug prototypes because of their remarkable chemical diversity [10]. Numerous efforts have been made to isolate novel compounds from medicinal plants. Currently, more than 20% of recently approved anticancer chemotherapeutic agents are derived from or related to NPs [11,12]. In addition, natural compounds with promising anticancer activities are apoptosis-inducing molecules [13,14,15]. In developing nations, traditional and folk medicinal practices, including the use and consumption of native medicinal plants, are still prevalent, especially in far-flung areas [16,17,18]. Therefore, investigations on NPs in the context of drug discovery may provide ethnomedicinal validation of the use of these plants while contributing to the drug discovery pipeline.

Indole moieties are active components of alkaloidal NPs. For example, aspidosperma–aspidosperma bisindole alkaloids represent a fascinating class of natural compounds with significant pharmacological potential, contributing to ongoing research on anticancer drug discovery and development. These bisindole alkaloids are characterized by their unique chemical structure, consisting of two indole rings linked together [19,20]. Bisindole alkaloids such as ervachinine A, taberdivarines, and voacalgine exhibit cytotoxicity against breast carcinoma, myeloid leukemia, colon adenocarcinoma, and lung carcinoma cell lines [21,22,23]. Meanwhile, the *Vinca* alkaloids vincristine and vinblastine serve as the best examples of the applications of bisindole alkaloids in the clinical setting [24,25].

**Figure 1:**
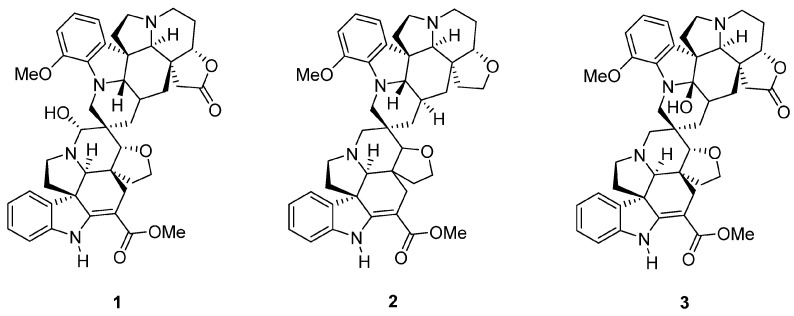
Spirobisindole alkaloids globospiramine (**1**), deoxyvobtusine (**2**), and vobtusine lactone (**3**) from the medicinal plant *Voacanga globosa*.

Relevant to this study, medicinal plants are among the most documented origins of bioactive NPs. *Voacanga globosa*, an endemic medicinal plant in the Philippines traditionally used for cancer, infection, and ulcer has been reported to contain the biologically active spirobisindole alkaloids globospiramine (**1**), deoxyvobtusine (**2**), and vobtusine lactone (**3**). These alkaloids exhibit anti-HIV, anti-*Mycobacterium tuberculosis*, and anticholinesterase properties [26,27]. The extracts and fractions of *V. globosa* have been investigated for their cytotoxic and antimicrobial activities. However, there is a clear gap for exploring the responsible phytoconstituents [28,29]. Additionally, the cytotoxic properties of phytoconstituents **1**–**3** have previously been investigated only using promyelocytic and lymphocytic cell lines to assess antiviral activity [27]. Herein, we demonstrate their biological activities against cancer cells and report the in vitro cytotoxic and antiproliferative activities of *V. globosa* alkaloids **1**–**3** (Figure 1) by performing apoptotic investigations into the most active alkaloid derivative globospiramine (**1**) using a combination of cell-based assays, network pharmacology, and in silico simulation experiments.

## 2. Materials and Methods

### 2.1. Test Compounds and Culture Conditions

The test compounds globospiramine (**1**), deoxyvobtusine (**2**), and vobtusine lactone (**3**) were isolated and purified from air-dried *V. globosa* leaves, collected from the Quezon Province, Luzon, the Philippines, in June 2006. A voucher specimen (USTH5015) consisting of the leaves, fruits, and stems of the plant was submitted to and deposited at the University of Santo Tomas Herbarium (USTH), and authentication and identification were carried out by the in-house botanist/plant systematist (Dr. Grecebio Jonathan Alejandro).

The isolation protocol was based on previously reported methods [26]. The pure compounds were stored in a −40 °C refrigerator until use. Alkaloids **1**–**3** were dissolved in dimethyl sulfoxide (DMSO) to obtain the desired concentrations. For the in vitro experiments, 0.5–1% DMSO was used. The test concentrations for subsequent viability assays (MTT and CellTiter-Blue) ranged from 333 μg/mL to 1.9 × 10^−3^ μg/mL. Cell cultures were grown in media with supplementation based on previously reported protocols [15,30].

### 2.2. In Vitro Cytotoxicity and Antiproliferative Activity Assessment

#### 2.2.1. MTT Assay

For the initial screening of the cytotoxicity of alkaloids **1**–**3**, assessment was performed using the non-tumorigenic cell line L929 and cancer cell lines KB3.1 (or HeLa), A431, MCF-7, PC-3, SKOV-3, and A549 using the 3-(4,5-dimethyl-2-thiazolyl)-2,5-diphenyl-2*H*-tetrazolium bromide (MTT) (Sigma-Aldrich, Darmstadt, Germany) assay. In brief, cells (approximately 50,000 cells/well) were treated with test alkaloids for 24 h. MTT was added to the cell suspension, followed by 4 to 5 h of incubation, following previously reported methods [30,31]. The half-maximal inhibitory concentration (IC_50_) was calculated on the basis of the absorbance (595 nm) readings in the microplate reader. For comparison, the positive control epothilone B was used [15].

#### 2.2.2. CellTiter-Blue^®^ Assay

Antiproliferative assessment of alkaloids **1**–**3** was performed using the CellTiter-Blue^®^ assay (Promega, Mannheim, Germany) based on the protocol set by Krauth et al. [32] and Otgon et al. [33]. Approximately 10,000 cells per well were seeded in 96-well plates with a 24 h incubation time. Test alkaloids were then added after washing the medium, and the cells were cultured at 37 °C. CellTiter-Blue^®^ reagent was added and allowed cell absorption for 2 h. Readings obtained from the microplate reader were analyzed, and the GI_50_ (concentration where there is 50% reduction in cellular growth compared with untreated control cells) was calculated using GraphPad Prism 9 (GraphPad software, San Diego, CA, USA). Imatinib was utilized as the positive drug control [34].

#### 2.2.3. SRB Viability Assay

The most active alkaloid **1** was subjected to a sulforhodamine B (SRB) viability assay to assess its cytotoxic effects on triple-negative breast cancer (TNBC) cell lines based on previously reported methods [35]. The cells in 96-well plates were allowed to adhere overnight. The cells were then fixed and stained after treatment with globospiramine (**1**) and controls for 48 h. This is the standardized protocol for cells that grow more slowly like receptor-deficient TNBC cells. Absorbance was determined using a SpectraMax plate reader (Molecular Devices, San Jose, CA, USA). Treatment concentrations were compared with vehicle control (0.5% DMSO)-treated cells to measure differences in cellular density. To generate the concentration–response curve for globospiramine (**1**), an initial concentration of 10 µM was used and diluted two-fold (12 times). For paclitaxel, 10 µM was used for the initial concentration, and 1 µM was used for combretastatin A4. In both drug controls, this initial concentration was diluted by 50% (12 times). The concentration resulting to 50% decline in cell density was reported as the IC_50_ and computed via non-linear regression analysis in GraphPad Prism 9. For positive controls, paclitaxel and combrestatin A4 were used.

### 2.3. Apoptosis Assays

#### 2.3.1. Caspase–Glo^®^ 3/7 Apoptosis Assay

The caspase–Glo^®^ 3/7 assay was performed according to the manufacturer’s instructions (Promega, Madison, WI, USA). In brief, A549 cells were seeded in a 96-well plate at a density of 10,000 cells/well. Cells were then treated with globospiramine (**1**) 6 h after seeding. Menadione sodium bisulfite (100 µM) and DMSO were used as positive and negative (vehicle) controls, respectively. Following 20 h of incubation, the cells were centrifuged at 2500× *g* for 5 min, and 20 µL of the resulting supernatant was transferred to a clean, opaque white 96-well plate. Reconstituted caspase–Glo^®^ 3/7 reagent was then added, and the plates were incubated at room temperature with shaking for 2 h. Luminescence readings were recorded using a FLUOstar Omega Microplate Reader (BMG Labtech, Offenburg, Germany).

#### 2.3.2. CellEvent^TM^ Caspase 3/7 and TMRM Staining

A549 cells were seeded at a density of 10,000 cells/well in a clear, flat-bottom 96-well plate. Treatment with globospiramine (0.1, 0.3, 1, 10, and 30 µM) was performed 6 h after seeding and incubated further at 37 °C and 5% CO_2_ for 20 h. Menadione sodium bisulfite (100 µM) and DMSO were used as positive and negative (vehicle) controls, respectively. Cells were then stained with CellEvent^TM^ Green Detection Reagent (Invitrogen) to confirm caspase 3/7 activity, tetramethylrhodamine methyl ester (TMRM; Invitrogen) to assess mitochondrial membrane potential, and Hoechst 33342 (Molecular Probes, Inc., Eugene, OR, USA) for nuclear visualization. Fluorescence imaging was performed 1 h after staining, and the images were subsequently analyzed using ImageJ (v1.54h) software. The percentage of apoptotic cells was computed as the ratio between the apoptotic cell count and the total nuclear count.

### 2.4. Western Blot Analysis

To determine the levels of BCL-2, full-length PARP-1, cleaved PARP-1 cleaved caspase 3, and α-tubulin in A549 cells, proteins were extracted and analyzed as follows: In brief, A549 cells (2 × 10^5^ cells/well) were cultured in 6-well plates. After 20 h of incubation, the cells were treated with DMSO or various concentrations (0.2, and 0.1 µM of globospiramine (**1**), respectively). Treatment incubation lasted for 36 h, which was heavily based on optimization and factors including protein (cleaved caspase 3 and PARP) collection and assay sensitivity, followed by washing the cells twice with ice-cold PBS and solubilized with a radioimmuno-precipitation assay (RIPA) buffer containing cocktail protease inhibitors and phosphatase inhibitors (0.02% NaF, 0.5% NaVO_4_, and 5% Na_4_P_2_O_7_). Protein concentrations in cell lysates were determined using the Bradford protein assay (BioRad). Equal amounts of protein (40 µg) per sample were cooked and pipetted into the gel, separated through gel electrophoresis, and electroblotted onto a polyvinylidene difluoride (PVDF) membrane. Blots were incubated in PBS containing 0.1% Tween (PBST) (pH 7.6) and 5% BSA. The cut-up membranes were then incubated with specific primary antibodies overnight and then incubated with their specific secondary antibodies. After washing with PBST, the blots were developed using NBT/BCIP before exposure to photographic films. The following antibodies were used: anti-cleaved PARP1 antibody (ab32064), anti-PARP1 antibody (ab191217), cleaved caspase 3 (#9661), BCL-2 antibody (AF0769), and α-tubulin antibody (1224-1AP). Analysis of the band intensity was conducted using ImageJ (v1.54h) software.

### 2.5. HeLa Cell Membrane Permeability Assay

To test for membrane permeability, HeLa cells were treated with 0.3 µM (IC_50_) and 3 µM (10× IC_50_) globospiramine (**1**) for 6 and 24 h. Cells were scraped and mixed with 1:1 Trypan Blue. Cell number was obtained by counting the internally dyed vs. non-internally dyed cells using a Countess 3 automated cell counter. Viability was shown as a percentage of the total in which the dyed cells were defined as dead cells and non-dyed cells were defined as live [36]. Phase-contrast microscopic images were generated using a Leica DM IL LED Inverted Microscope (Leica Microsystems, Wetzlar, Germany).

### 2.6. Network Pharmacology Analysis

#### 2.6.1. Prediction of Target Genes

The SMILES format of globospiramine (**1**) was obtained by drawing its structure in ChemDraw 18.1 and confirmed in PubChem (https://pubchem.ncbi.nlm.nih.gov/ (accessed on 7 July 2023)). It was then converted into a mol2 file using the Avogadro (1.2.0) software. To identify putative target genes, the mol2 file was used in SWISS Target Prediction (*Homo sapiens* only) (http://www.swisstargetprediction.ch (accessed on 7 July 2023)), while the SMILES notation was uploaded to the PharmMapper database (https://www.lilab-ecust.cn/pharmmapper/index.html (accessed on 7 July 2023)).

The potential therapeutic target genes associated with the most sensitive cancer cell lines were identified by inputting the name of the cell line in DisGeNET (https://www.disgenet.org/ (accessed on 7 October 2023)) and GeneCards (https://www.genecards.org/ (accessed on 7 October 2023)), with scores ≥ 0.8 and > 10, respectively, as threshold values based on the methods of Shang et al. [37]. For standardization, the UniProt IDs of proteins were utilized for both disease-associated genes and globospiramine targets.

#### 2.6.2. Protein–Protein Interaction Analysis

Common genes between globospiramine (**1**) targets and genes associated with sensitive cancer cell lines were determined using the online bioinformatics platform JVENN (https://www.bioinformatics.com.cn/static/others/jvenn/example.html (accessed on 7 October 2023)) for Venn diagram analysis [38]. The protein–protein interactions (PPI) of common genes were then visualized in the STRING database (https://string-db.org/ (accessed on 7 October 2023)) with a confidence score > 0.9 (high confidence), and species selection was limited to *Homo sapiens*. Further PPI analysis was carried out in Cytoscape (3.10.1) to yield the top 10 potential core genes based on the Maximal Clique Centrality (MCC) algorithm [39,40].

#### 2.6.3. GO and KEGG Pathway Enrichment Analyses

Gene Ontology (GO) and the Kyoto Encyclopedia of Genes and Genomes (KEGG) pathway analyses were performed using WebGestalt (https://www.webgestalt.org/option.php (accessed on 9 October 2023)). The putative target pathway, which was identified on the basis of the enrichment ratio, was visualized in an available database (https://www.genome.jp/kegg/ (accessed on 9 October 2023)). Putative targets for molecular docking were selected on the basis of the KEGG map [37].

### 2.7. Molecular Docking to MAPK14 (p38α)

Globospiramine (**1**) was used as the ligand. The structure was drawn in ChemDraw (18.1) and saved in the SMILES format for conversion into mol2 in Avogadro (1.2.0). The following six protein targets were fetched through their Protein Data Bank (PDB) IDs (https://www.rcsb.org/ (accessed on 11 December 2023)) and processed in UCSF Chimera (1.17.3): AKT1 (PDB ID: 4GV1), AKT2 (PDB ID: 2JDR), PIK3CA (PDB ID: 5XGJ), MAPK14 (PDB ID: 3FLY), TNFR1 (PDB ID: 1EXT), and TNFR2 (PDB ID: 3ALQ). These proteins were prepared and minimized in UCSF Chimera (1.17.3) according to the procedure described in previous studies [41]. In brief, the co-crystallized structures and non-standardized atoms that were bound in the downloaded proteins were deleted. Minimization was performed following the steepest descent method with 100 steps at the default step size and the conjugate gradient method with 10 steps. Prepared proteins were formatted as PDB files. All molecular docking experiments were carried out using the UCSF Chimera software (1.17.3). The ligand in the mol2 file and the target protein in the PDB format were inputted into the software. Dock prep and molecular docking simulations were performed according to the flexible ligand into flexible active site protocol. Ten binding modes were predicted for each complex based on the BFGS algorithm and embedded via AutoDock Vina. The COACH algorithm was utilized for the generation of a grid that was manually created to encompass the binding pocket and key residues involved in the activity of the protein targets. To visualize and analyze the interactions between globospiramine (**1**) and the target proteins, the output files for both the ligand and the dock complexes were added to BIOVIA Discovery Studio software (4.1).

### 2.8. Molecular Dynamics (MD) Simulation

MD simulation studies using CUDA API technology with two NVIDIA graphics processing units (GPUs) were conducted using Desmond software through the graphical interface of Maestro (Desmond Molecular Dynamics System 6.4 academic version, D. E. Shaw Research (“DESRES”), New York, NY, USA, 2020. Maestro-Desmond Interoperability Tools, Schrödinger, New York, NY, USA, 2020). To generate the three-dimensional ligand/protein complex (MAPK14/globospiramine) embedded into an orthorhombic box filled with water molecules (TIP3P), the system builder provided by Desmond software was utilized [42,43]. Na^+^ and Cl^−^ ions were added to the biological system to reach a physiological concentration of monovalent ions of 0.15 M. The OPLS3 force field was utilized to perform the MD simulation [44]. The NPT ensemble class (constant number of particles, 300 K for the temperature, and 1.01325 bar for the pressure) was used for the simulation. The RESPA integrator (inner time step of 2.0 fs) was utilized to estimate the motion for bonded and non-bonded interactions within the short-range cutoff [45]. To maintain a constant temperature during the simulation, the Nosé–Hoover thermostat technique was employed [46], whereas the pressure was kept constant by utilizing the Martyna–Tobias–Klein method [47]. To compute long-range electrostatic interactions, the particle mesh Ewald technique (PME) was applied (van der Waals and short-range electrostatic interactions were fixed at 9.0 Å) [48]. Using the default procedure, which involved several constrained minimizations and MD simulations, the selected system was gradually relaxed and brought to equilibrium. The Desmond package’s simulation event analysis tools were employed to investigate the MD outputs generated during the MD simulation experiments, as previously reported [49].

### 2.9. Profiling of Drug-Likeness and Toxicity Risks

Globospiramine (**1**) in SMILE notation was submitted to the SwissADME website (http://www.swissadme.ch/ (accessed on 11 December 2023)), and drug-likeness was assessed on the basis of Veber’s rule. The pharmacokinetic profile was analyzed using the BOILED-Egg method. For toxicity risk prediction, the OSIRIS Property Explorer was used [50,51].

### 2.10. Statistical Analysis

Data analyses were performed using GraphPad Prism 9. For the apoptosis assays, an independent *t*-test was performed to compare the results for the treatment groups to those of the vehicle control. For quantified proteins in Western blot, all data results were analyzed using variance analysis, followed by Dunnett’s test for pairwise comparison. The statistical significance was defined as * *p* < 0.05, ** *p* < 0.01, *** *p* < 0.001, and **** *p* < 0.0001 for comparison with the vehicle control cells.

## 3. Results

### 3.1. Alkaloids **1**–**3**, Especially **1**, Exhibits In Vitro Cytotoxic and Antiproliferative Activities

The spirobisindole alkaloids globospiramine (**1**), deoxyvobtusine (**2**), and vobtusine lactone (**3**) from the traditional anticancer medicinal plant *Voacanga globosa* were evaluated for in vitro cytotoxicity and antiproliferative activities against tested mammalian tumorigenic and non-tumorigenic cell lines using MTT and CellTiter-Blue^®^ assays. Alkaloid **1** demonstrated the most potent cytotoxicity (IC_50_ = 0.01 to 0.22 µM) and antiproliferative activity (GI_50_ = 1.91 to 7.37 µM), while alkaloid **3** showed moderate cytotoxicity (IC_50_ = 2.32 to 10.64 µM) and antiproliferative activity (GI_50_ = 7.64 to 22.10 µM). Alkaloid **2** showed cytotoxicity to two cell lines (non-tumorigenic L929 and HeLa KB3.1). Compared to the positive control epothilone B, the cytotoxic and antiproliferative activities of the test alkaloids **1**–**3** were relatively lower (Table 1).

The in vitro potency of globospiramine (**1**) against the cell lines tested in Table 1 prompted us to further investigate its cytotoxic and antiproliferative effects on the triple-negative breast cancer (TNBC) cell lines HCC1806, HCC1937, MDA-MB-453, MDA-MB-231, and BT-549 using a SRB assay (Table 2). Alkaloid **1** exhibited higher IC_50_ values than the positive controls paclitaxel and combrestatin A4.

### 3.2. Globospiramine (**1**) Induces Caspase-Dependent Apoptosis in A549 Cells in a Concentration-Dependent Manner

**Figure 2:**
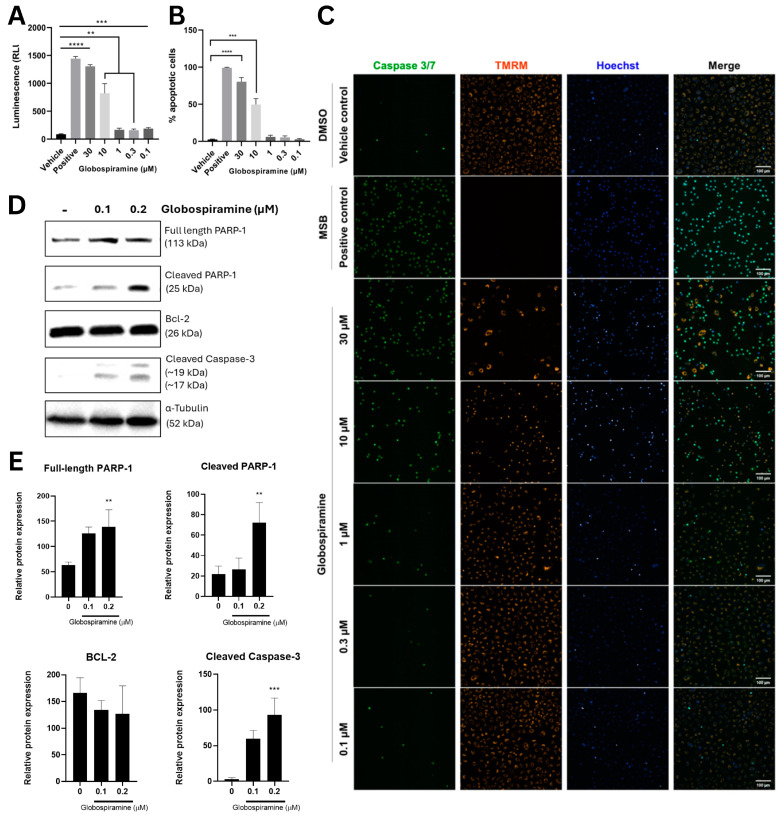
(**A**) Caspase–Glo 3/7 apoptosis assay demonstrated significantly increased normalized luminescence values relative to vehicle control (DMSO only) upon treatment of A549 cells with different concentrations of globospiramine (**1**) (30, 10, 1, 0.3, and 0.1 µM, respectively), suggesting pro-apoptotic bioactivity in a dose-dependent manner. (**B**) CellEvent apoptosis assay revealed similar trend with significant increase in % apoptotic cells for globospiramine (**1**) at concentrations of 30 µM and 10 µM vs. DMSO. (**C**) Fluorescence microscopic analysis showed that globospiramine (**1**) treatment also induced apoptosis in a concentration-dependent manner. A549 cells were treated with varying concentrations of **1** and the extent of apoptosis was visualized quantitatively using CellEvent Caspase 3/7 green detection reagent, co-stained with TMRM (mitochondrial integrity) and Hoechst 33342 (nucleus), 20 h post-treatment. DMSO and menadione bisulfate were used as vehicle and positive controls, respectively. Scale bars (100 µm) were included in the micrographs. (**D**,**E**) Western blot analysis of apoptotic proteins showed increased expression of cleaved PARP-1 and cleaved caspase 3. The protein ⍺-tubulin was used as internal control, whereas full-length PARP-1 and Bcl-2 were used to track possible mechanism of cell death regulation. Three replicates were performed for all experiments (** *p* < 0.01, *** *p* < 0.001, and **** *p* < 0.0001).

To gain initial insights into the mechanism of action of the most cytotoxic alkaloid globospiramine (**1**), we performed apoptosis assays (Caspase–Glo^®^ 3/7 and CellEvent^TM^ apoptosis assays). To investigate whether the cytotoxic concentration would trigger apoptosis in cancer cells, we selected a single cell line (A549) and investigated its sensitivity to globospiramine (**1**) in the context of apoptosis induction (Table 1). Accordingly, the Caspase–Glo^®^ 3/7 assay demonstrated that globospiramine (**1**) treatment significantly induced apoptosis across all concentrations (30, 10, 1, 0.3, and 0.1 µM) tested compared with the vehicle control DMSO (Figure 2A). These data are further supported by the results of the CellEvent^TM^ assay, where similar dose-dependent trends were observed. However, unlike the previous assay, only 30 µM (*p* < 0.0001) and 10 µM (*p* < 0.001) demonstrated significant increases in the percentage of apoptotic cells compared to the vehicle control (Figure 2B). The accompanying fluorescence microscopy images reflect caspase 3/7 activity, as well as changes in mitochondrial membrane potential based on TMRM signal, upon treatment (Figure 2C).

Western blot analysis was also carried out to evaluate the effects of **1** on the expression of key proteins involved in the apoptotic pathway using A549 cells. The results corroborated with the caspase–Glo 3/7 apoptosis assay results and showed that low concentrations of **1** (0.1 and 0.2 µM) promoted the cleavage of caspase 3 and PARP-1, which are events indicative of cells undergoing apoptosis, after 36 h of exposure (Figure 2D). Globospiramine (**1**) at 0.2 µM significantly induced increased expression of cleaved PARP-1 (*p* < 0.01) and caspase 3 (*p* < 0.001) (Figure 2E).

### 3.3. Globospiramine (**1**) Did Not Cause Rapid Loss of Membrane Integrity in HeLa Cells

**Figure 3:**
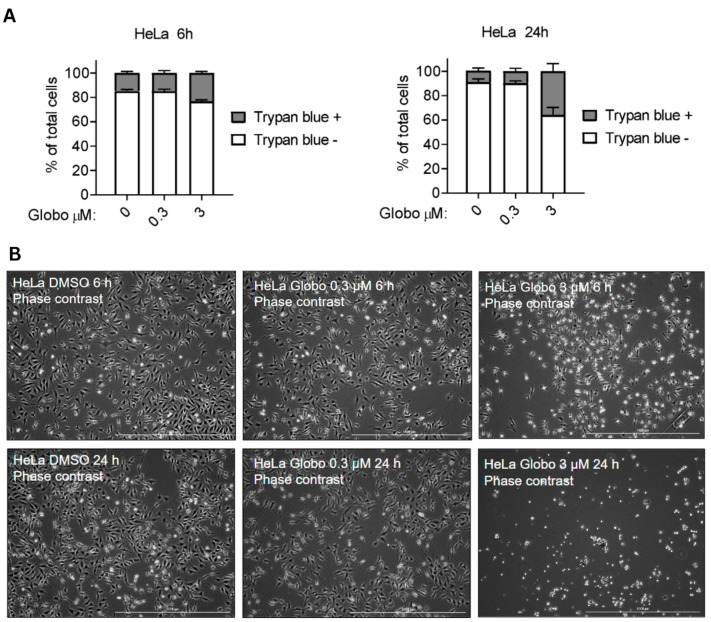
HeLa cell membrane permeability assay via Trypan blue diffusion was used to assess the putative membrane-compromising effects of globospiramine (**1**). Diffusion of Trypan blue dye inside cells (Trypan blue +) indicates a compromised membrane. Alkaloid **1** promoted compromised cell membranes in a relatively small proportion of cells compared with the results of in vitro cytotoxicity vs. HeLa cells. Three replicates were performed. (**B**) Phase-contrast microscopic analysis showed a similar trend, with observable compromised cell membrane at 3 µM after 24 h of exposure.

We also investigated whether globospiramine (**1**) may promote compromised cell membrane permeability using a Trypan blue diffusion assay on HeLa cells. Despite exposure to **1** at a high concentration (3 µM), only approximately 40% of cells showed compromised cell membrane permeability after 24 h of exposure (Figure 3A). This result was further supported by phase-contrast microscopic analysis (Figure 3B). These findings further confirmed the apoptotic activity of compound **1**, as rapid loss of membrane integrity is not considered to be a hallmark of apoptosis.

### 3.4. MAPK14 (p38α) as the Putative Molecular Target of Globospiramine (**1**) Based on Network Pharmacology and Molecular Docking

**Figure 4:**
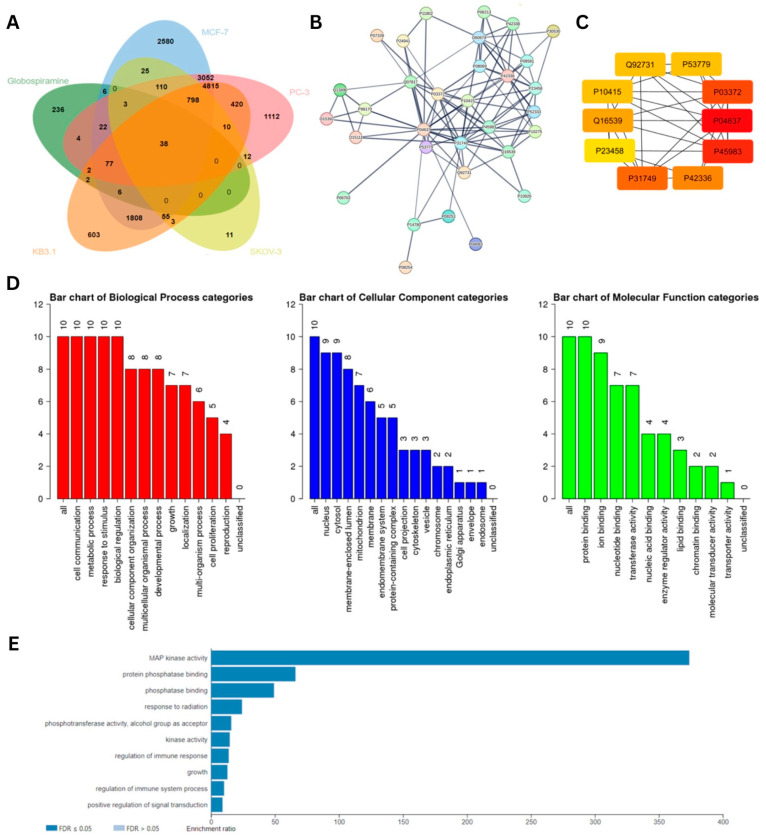
Results of bioinformatics-guided mining and PPI interaction analysis of potential molecular targets showing (**A**) the 38 common genes between globospiramine (**1**) targets and genes associated with the most sensitive cell lines, (**B**) the visualized PPI interactions from the STRING database, and (**C**) the 10 identified putative therapeutic target core genes in Cytoscape (3.10.1). Proteins were standardized using their UniProt IDs. (**D**) Characteristics of the 10 core genes based on Gene Ontology (GO) analysis in terms of their biological process (BP), cellular component (CC), and molecular function (MF). (**E**) Results of KEGG pathway enrichment analysis showing the MAPK pathway as the top target (*p*-value = 5.3491 × 10^−8^; FDR = 0.00010558).

To predict the molecular targets of **1**, we performed a series of network pharmacology analyses and molecular docking. A total of 391 putative targets of **1** were obtained from the SWISS Target Prediction database and PharmMapper. Based on the in vitro cytotoxic and antiproliferative activities of **1** (Table 1), cancer cell lines that are most sensitive to **1** were utilized in bioinformatics-driven gene mining in the DisGeNET and GeneCards databases (Supplementary Table S1). The UniProt IDs of the gene targets were subsequently inputted into the JVENN data analysis and visualization online platform, and the results revealed a total of 38 intersecting genes across the cell lines and globospiramine (**1**) (Figure 4A).

The results of visualization and further protein–protein interaction (PPI) analysis of these common genes, which was performed in the STRING database, demonstrated a total of 32 nodes and 103 edges with high confidence (confidence value > 0.9). Further PPI analysis performed by importing the STRING output file into Cytoscape (3.10.1) identified the top 10 genes based on centrality, as computed by the Maximal Clique Centrality (MCC) algorithm (Figure 4B,C). The UniProt IDs of the proteins were used for standardization, and the corresponding protein names or symbols are as follows: TP53 (P04637), MAPK8 (P45983), ESR1 (P03372), AKT1 (P31749), PIK3CA (P42336), MAPK14 (Q16539), ESR2 (Q92731), MAPK10 (P53779), BCL2 (P10415), and JAK1 (P23458). To further characterize the central genes, they were subjected to Gene Ontology (GO) analysis (Figure 4D). GO analysis revealed the predicted biological processes (BPs), cellular components (CPs), and molecular functions (MFs) associated with the central genes. BPs were associated with cell communication, metabolic processes, responses to stimuli, and biological regulation. In terms of CP, 9 out of 10 genes were found in the nucleus and cytosol. Interestingly, all targets were predicted to be involved in protein binding for their MF.

The same set of core genes underwent Kyoto Encyclopedia of Genes and Genomes (KEGG) pathway enrichment analysis. The mitogen-activated protein kinase (MAPK) pathway emerged as the top pathway with an enrichment ratio of >350 (FDR ≤ 0.05) (Figure 4E). Upon analysis of the MAPK KEGG map, the tumor necrosis factor (TNF) signaling pathway was selected for further KEGG pathway analysis because of its involvement in promoting apoptosis after exposure to cytotoxic drugs (Supplementary Figure S1A). Therefore, the KEGG map of the TNF signaling pathway was analyzed, and the MAPK and PI3K-AKT signaling pathways were implicated downstream (Supplementary Figure S1B). From these pathways, molecular targets, specifically those that have been reported to play crucial roles in cancer progression and poorer disease outcomes, were utilized for molecular docking.

**Figure 5:**
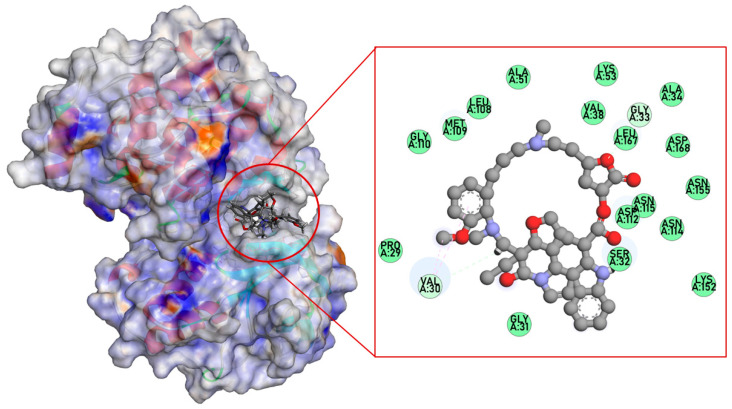
Dock pose of globospiramine vs. putative molecular target MAPK14 (p38α).

To investigate the molecular interactions of globospiramine (**1**) inside the binding pockets of selected target proteins, molecular docking simulations were performed. Six targets from the MAPK, TNF, and PI3K-AKT signaling pathways were utilized. Globospiramine (**1**) showed the highest binding affinity for MAPK14 (p38α) (BE = −9.8 kcal/mol) (Table 3). It was bound to MAPK14 (p38α) via *pi*-alkyl, alkyl, and C-H bonds with Val30, as well as with Gly33 (Table 3, Figure 5). Despite the presence of only two interacting amino acid residues, alkaloid **1** conferred better BE than the positive control.

### 3.5. Molecular Dynamics Simulations Showed Stability of Globospiramine (**1**) Inside the MAPK14 or p38α Binding Domain

**Figure 6:**
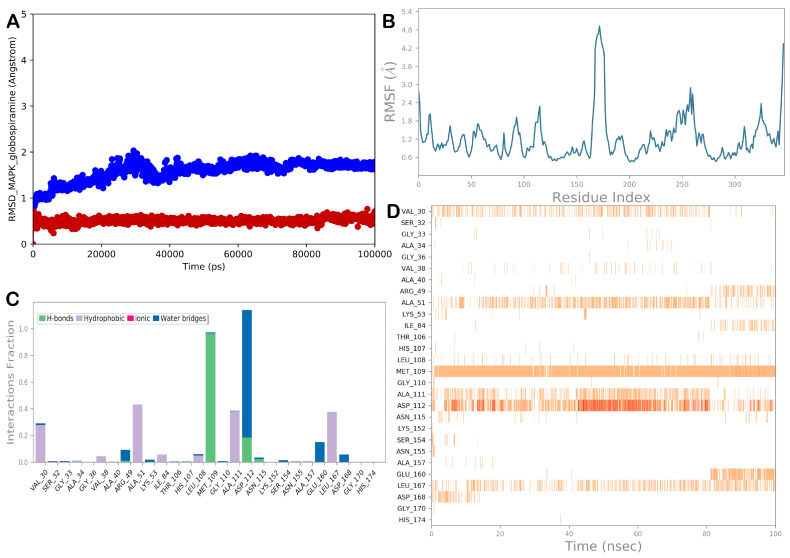
(**A**) Evaluation of the RMSD for the selected complex (the blue line represents the protein, while the red line represents the ligand). (**B**) RMSF evaluation of the biological system MAPK/globospiramine after 100 ns of MD simulation. (**C**,**D**) Globospiramine was monitored during the entire MD trajectory. There are four different forms of interactions: blue represents water–bridge contacts, magenta represents ionic interactions, gray represents hydrophobic interactions, and green represents H-bonds. The stacked bar charts are normalized as they progress throughout the trajectory. For example, a score of 0.7 suggests that a certain contact is kept 70% of the time during simulation. Values larger than 1.0 could happen if a protein residue uses the same subtype to bind with the ligand more than once. The diagram below provides a temporal explanation of the main interactions. The results show the residues that interact with the ligand in each trajectory frame. A deeper orange indicates several interactions between certain residues and the ligand. Images were produced using software tools from Desmond and Maestro (Maestro, Schrödinger LLC, release 2020-3).

To predict the molecular targets of **1**, we performed a series of network pharmacology analyses, and to provide a comprehensive overview of the binding mode of globospiramine to the most probable target, MAPK14, we conducted MD simulation studies. Apart from visually examining the generated trajectory, the trajectory was evaluated using the computation of RMSD and RMSF, as well as the analysis of the dynamic ligand interaction graphs. Overall, the MD simulation results demonstrated that when complexed with the chosen proteins, globospiramine exhibited small RMSD values and slight variations in the proteins, as shown by the RMSF (Figure 6). In particular, hydrophobic contacts with Val30 were evident during the simulation. In addition, other hydrophobic contacts with Ala51, Ala111, and Leu167 were detected. A strong network of polar contacts (H-bonds) with the backbone of Met109 and the side chain of Asp112 was observed, although the H-bond with Asp112 was sometimes water-mediated.

### 3.6. Globospiramine (**1**) Was Predicted to Have No Toxicity Risks and Favorable Drug-Likeness

In silico pharmacokinetic and toxicity profiling of globospiramine (**1**) was also performed to predict its drug-likeness. The results showed that globospiramine (**1**) conferred no toxicity risks and favorable drug-likeness based on its physicochemical properties according to Veber’s rule (Table 4). The compound was also predicted to have high gastrointestinal (GI) absorption and non-blood–brain barrier permeation.

## 4. Discussion

Our findings demonstrate the uncontested relevance of natural products, especially alkaloids, in anticancer drug discovery. The in vitro cytotoxic and antiproliferative activities and mechanisms of spirobisindole alkaloids, especially globospiramine (**1**), from the traditional anticancer plant *Voacanga globosa* provide scientific evidence for the ethnomedicinal and traditional claims on the potency of the plant against malignant tumors. This also strengthens previously reported findings wherein *V. globosa* extracts and fractions were reported to confer in vitro cytotoxic and apoptosis-inducing properties [28,29]. In addition, this study contributes to the growing pharmaceutical properties such as the antiviral, antimicrobial, and anticholinesterase activities of *V. globosa* and its phytoconstituents [26,27,28,54].

This study also highlights that globospiramine (**1**) is the most biologically active phytoconstituent against a panel of cancer cell lines in vitro. This compound belongs to the class of bisindole alkaloids, such as vinblastine and vincristine, which are generally known for their anticancer properties. Structurally, the rearrangement and oxidative functionalization of the side skeleton, which may incorporate carbonyl, methoxyl, and hydroxyl groups at varying spatial positions, have increased the diversity of indole alkaloids, which is translated into their myriad of biological activities [24,25]. The indole moiety is also considered to be the molecular framework responsible for the pharmacological activities of several currently available drugs, such as phosphodiesterase-5 inhibitors, antilipemic agents, and serotonin receptor agonists [55]. However, despite the occurrence of indole structures in all tested alkaloids, the characteristic presence of carbinolamine in C-3 for **1** may have contributed to its increased potency [26]. Recently, cytotoxic aspidosperma–aspidosperma spirobisindole alkaloids from *Tabernaemontana pachysiphon* have been reported. Given that globospiramine **1** showcases the same aspidosperma–aspidosperma skeleton, comparing their anticancer activities may shed light on their structure–activity relationships. Globospiramine (**1**) demonstrated better IC_50_ against A549 (IC_50_ = 0.18 uM) and MDA-MB-231 (IC_50_ = 0.387 uM) cells than all *T. pachysiphon* alkaloids (IC_50_ > 8 uM) despite their high structural similarity. Some minor differences include the distinct presence of methoxy and the different spatial positions of hydroxylation in globospiramine (**1**), which may have contributed to its robust cytotoxicity [56].

To elucidate the mechanism of action of globospiramine (**1)**, its pro-apoptotic activities were investigated. In the context of cancer therapy, the ability of tumor cells to evade apoptosis has been implicated in metastasis and the development of drug resistance. In fact, combining apoptosis-inducing alkaloids with other standard chemotherapies has been suggested by several studies to combat chemoresistance [57,58]. Our results indicated that globospiramine (**1**) can mediate robust apoptotic activity, as evidenced by the activation of executioner caspases 3 and 7, as well as the cleavage of PARP-1. As soon as apoptosis is signaled, caspases 3 and 7 are cleaved and activated and subsequently cleave their target proteins, which are necessary for normal cellular physiology. Dismantling these proteins leads to apoptosis [59,60]. In contrast, PARP-1 is a cellular substrate of caspases, including caspases 3 and 7. Therefore, the cleavage of PARP-1 is considered an apoptosis hallmark [61,62]. Meanwhile, the increased expression of full-length PARP-1 was evident with 0.2 uM of globospiramine. Increased expression of PARP-1 leads to increased DNA repair of damage carried out by ROS for genome stability [63]. In different cancer cell lines, PARP-1 expression was increased in hepatocellular carcinoma, and in non-small-cell lung cancer cell lines, it was resistant to cisplatin [64,65]. Chemotherapeutic agents promote DNA damage in cells, so the significant presence of PARP-1 in higher doses of the compound possibly implies that the genomic instability elicited increased expression of the enzyme [66].

The apoptosis-inducing property of **1** was further confirmed through TMRM staining for mitochondria and Trypan Blue diffusion assay for cell membrane permeability. The TMRM signal disappears when there is a loss of mitochondrial membrane potential, an indicator that cells are undergoing apoptosis due to the loss of well-functioning mitochondria [67]. Additionally, **1** did not induce extensive or rapid loss of cell membrane integrity. Apoptosis, in general, is characterized by the packaging of membrane-bound vesicles as dying cells dismantle; thus, it is expected that our compound did not elicit compromised membrane permeability in cancer cells as it underwent apoptosis [68]. Yet, to ascertain our phase-contrast microscopic analysis results, flow cytometry, scanning electrochemical microscopy, and other more advanced methods are likewise recommended. In this way, there would be more adequate evidence to show the effects of globospiramine on cell morphology and membrane integrity. Overall, it is important to note that the apoptosis investigations were only carried out for A549 cells; therefore, further analysis of the effects of globospiramine (**1**) on other cell lines is warranted to ascertain its apoptosis-inducing activities against several types of cancer.

Referring back to our in vitro cytotoxicity data, the sensitivities of cancer cells to globospiramine (**1**) differ depending on the type of cancer. This is commonly observed as some pathways and proteins are either overexpressed (upregulated) and/or repressed (downregulated) depending on the cancer type. In fact, most drugs were reported to have varying effects even to distinct cancer cell line subtypes [69,70]. In our bioinformatics and in silico experiments, several cancer-associated genes commonly expressed in a wide variety of cancer cell lines were investigated. Of note, our molecular docking and MD simulation output indicated relevant interactions within the selected binding site, suggesting the potential of globospiramine (**1**) to act as an inhibitor of MAPK14 or p38α based on the affinity of the compound to the protein, the stability of the biological system, and the significant number of contacts it made inside the chosen binding site throughout the course of the 100 ns MD simulations. The MAPK pathway is among the common pharmacologic targets in cancer therapy, and this could explain why globospiramine (**1**) seems to have a general cytotoxicity against cancer cells. In the context of anticancer drug discovery, the MAPK14 (p38α), a protein in the MAPK cascade, has a wide array of functions in cell cycle regulation, proliferation, tumor aggressiveness, and cell death. The inhibition of p38α has been considered an emerging mechanism of action for anticancer drugs [71,72]. In addition, MAPK14 has been reported to be activated by environmental stressors, including drug induction, and was investigated to discover compounds with activities against A549 cells. In non-small-cell lung cancer, there is overexpression of elements, including MAPK14, in the MAPK signaling pathway, thereby highlighting the need to inhibit the enzyme [73,74]. In the study of Mesquita et al. [75], p38α inhibition promoted the G_0_/G_1_ arrest of gastric tumor cells, leading to the activation of effector caspases 3 and 7 and, eventually, apoptosis. In our findings, the effect of **1** on different sensitive cell lines, including HeLa cells, may have also promoted the deregulation of the MAPK14 pathway, thereby converging on common apoptotic machinery. Our results also corroborate earlier reports showing the therapeutic effects of small molecules on cancer cell proliferation and apoptosis through the MAPK14 pathway [76,77,78]. The modulation of the p38 MAPK/JNK pathway has also been implicated in the antagonistic mechanism of a TNF-α-derived nanodrug against prostate cancer, which promotes caspase-dependent apoptosis [79]. Interestingly, the in silico binding activity of globospiramine (**1**) to MAPK14 may also explain its relatively weaker in vitro cytotoxicity against triple-negative breast cancer cell lines, especially MDA-MB-231. According to Duzgun et al. [80], MDA-MB-231 lacks replicative immortality involving the MAPK14 pathway. Therefore, our in silico findings warrant further investigations into the effects of **1** on the predicted target MAPK14.

## 5. Conclusions

In summary, our study provided scientific evidence for the ethnomedicinal use of *Voacanga globosa* extracts against cancer due to its bioactive phytoconstituents globospiramine (**1**), deoxyvobtusine (**2**), and vobtusine lactone (**3**). We also reported the cytotoxic mechanisms of action of **1**, which include the induction of apoptosis vs. A549 cells and potential MAPK14 inhibition. Its effect on the HeLa cell membrane integrity was also elucidated. Globospiramine (**1**) is hereby highlighted as an interesting scaffold for the discovery of new anticancer agents.

## Figures and Tables

**Table 1:** In vitro cytotoxicity and antiproliferative activity of alkaloids **1**–**3** on cell lines of different cancer types using MTT and CellTiter Blue assays.

Cell Lines	Test Compounds			Positive Controls	
	Globospiramine (1)	Deoxyvobtusine (2)	Vobtusine Lactone (3)	Epothilone B	Imatinib
Cytotoxicity IC_50_ (µM) | MTT Assay					
L929	0.05	40.93	4.64	0.001	-
KB3.1 (HeLa derivative)	0.22	11.60	4.64	3.27 × 10^−5^	-
A431	0.18	>50	10.23	0.0001	-
MCF-7	0.11	>50	5.46	9.55 × 10^−5^	-
A549	0.18	>50	10.64	4.23 × 10^−5^	-
PC-3	0.01	>50	2.32	5.87 × 10^−5^	-
SKOV-3	0.08	>50	11.05	0.0001	-
Antiproliferative activity GI_50_ (µM) | CellTiter Blue Assay					
HUVEC	7.37	>50	22.10	-	14.87
K-562	1.91	>50	7.64	-	0.14

(-) = not determined; DMSO served as negative control and showed no bioactivity. Three replicates were performed.

**Table 2:** In vitro cytotoxicity screening of globospiramine (**1**) against TNBC cell lines using SRB assay.

Test Compounds	Cell Lines IC_50_ (uM)					
	HCC1806	HCC1937	MDA-MB-453	MDA-MB-231	BT-549	HeLa
Globospiramine (**1**)	0.154	0.282	0.546	0.387	0.470	0.300
Paclitaxel	0.014	>0.16	0.002	0.028	>0.16	-
Combrestatin A4	>0.1	>0.1	>0.1	0.038	>0.1	0.003

(-) = not determined. Three replicates were performed.

**Table 3:** Binding affinities expressed as binding energies (BE) and interactions between globospiramine (**1**) and target proteins.

Targets	BE (kcal/mol)	Interactions	BE (kcal/mol) Positive Control
RAC-α serine/threonine-protein kinase (AKT1)	−9.2	Thr160, Thr291, Asn279 (H-bond), Phe442 (*pi*-*pi* T-shaped), Glu234 (salt bridge, C-H bond), Gly159 (C-H bond), Asp292 (*pi*-anion), Leu295 (*pi*-alkyl)	−8.6 ^a^
RAC-β Serine/Threonine-Protein Kinase (AKT2)	−2.9	Ly160 (H-bond), Asp440, Phe163, Glu279 (attractive charge), Glu279 (C-H bond)	
Phosphatidylinositol 4,5-bisphosphate 3-kinase catalytic subunit α isoform–chain A (PIK3CA)	−8.5	Asp926, Arg281 (attractive charge, *pi*-cation), Arg852, Val851 (alkyl)	−8.7 ^b^
Mitogen-activated protein kinase 14 (p38α)	−9.8	Val30 (alkyl, *pi*-alkyl, C-H bond), Gly33 (C-H bond)	−8.1 ^c^
Tumor necrosis factor Receptor 1 (TNRF1)	−7.9	Val95, Arg92, Ala61 (alkyl, *pi*-alkyl), Ser108 (C-H bond)	−9.9 ^d^
Tumor necrosis factor Receptor 2 (TNRF2)	−9.0	Lys120 (H-bond), Pro117 (alkyl, *pi*-alkyl, C-H bond), Arg119 (alkyl, *pi*-alkyl), Arg122 (*pi*-cation), Leu118 (unfavorable positive-positive, vdW)	−6.5 ^e^

BE = binding energy; superscripts a to f correspond to positive controls, which inhibit or modulate the targets: ^a^ ipatasertib, ^b^ copanlisib, and ^c^ ralimetinib; ^d^ ZINC ID: ZINC02968981; ^e^ ZINC ID: ZINC72321887. Positive controls a-c are known target inhibitors, while d-e were selected based on previous findings due to the lack of clinically approved controls [52,53].

**Table 4:** In silico evaluation of the drug-likeness, pharmacokinetic profile, and toxicity risks of globospiramine (**1**).

Physicochemical Properties	Accepted/Threshold Values	Predicted Values for
Rotatable bonds	≤ 10	3
TPSA (Å^2^)	≤140	113.04
Drug-likeness based on VR	Yes (no violation)	
Pharmacokinetic Profile		
GI absorption	High	
BBB-permeant	No	
Toxicity Risks		
Mutagenicity	None	
Tumorigenicity	None	
Irritant toxicity	None	
Reproductive toxicity	None	

VR = Veber’s rule; TPSA = topological polar surface area; GI = gastrointestinal; BBB = blood–brain barrier.

## Data Availability

The original contributions presented in this study are included in the article/Supplementary Material; further inquiries can be directed to the corresponding author/s.

## Supplementary Materials

The following supporting information can be downloaded via this link: https://www.mdpi.com/article/10.3390/cells13090772/s1.
