# Supplementary material for: Globospiramine from Voacanga globosa Exerts Robust Cytotoxic and Antiproliferative Activities on Cancer Cells by Inducing Caspase-Dependent Apoptosis in A549 Cells and Inhibiting MAPK14 (p38α): In Vitro and Computational Investigations
Source: Cells. 2024 Apr 30;13(9):772. doi: 10.3390/cells13090772 (PMC11082999; doi:10.3390/cells13090772)
Supplement: Supplementary file 1 [file cells-13-00772-s001.zip › cells-2931030-supplementary.pdf]

SUPPLEMENTARY MATERIAL FOR:

Globospiramine from *Voacanga globosa* Exerts Robust Cytotoxic and Antiproliferative Activities on Cancer Cells by Inducing Caspase-Dependent Apoptosis in A549 Cells and Inhibiting MAPK14 (p38α): In Vitro and Computational Investigations

Joe Anthony H. Manzano [1,2,3], Elian Angelo Abellanosa [3], Jose Paolo Aguilar [2], Simone Brogi [4] Chia-Hung Yen [5,*], Allan Patrick G. Macabeo [3,6,*], and Nicanor Austriaco [2,7,*]

[1] The Graduate School, University of Santo Tomas, España Blvd., Manila 1015 Philippines

[2] UST Laboratories for Vaccine Science, Molecular Biology and Biotechnology, Research Center for the Natural and Applied Sciences, University of Santo Tomas, España Blvd., Manila 1015 Philippines

[3] Laboratory for Organic Reactivity, Discovery, and Synthesis (LORDS), Research Center for the Natural and Applied Sciences, University of Santo Tomas, España Blvd., Manila 1015 Philippines

4 Department of Pharmacy, University of Pisa, Via Bonanno 6, 56126, Pisa, Italy

[5] Graduate Institute of Natural Products, College of Pharmacy, Kaohsiung Medical University, Kaohsiung 80708, Taiwan

[6] Department of Chemistry, College of Science, University of Santo Tomas, España Blvd., Manila 1015 Philippines

[7] Department of Biological Sciences, College of Science, University of Santo Tomas, España Blvd., Manila 1015 Philippines

* Correspondence: chyen@kmu.edu.tw (C.-H.Y.), agmacabeo@ust.edu.ph (A.P.G.M.), naustriaco@ust.edu.ph (N.A.); Tel.: (+632-74061611 ext. 4056)

LIST OF SUPPLEMENTARY MATERIALS

Supplementary Table S1 **Page 3**
Supplementary Figure S1 **Page 4**

Supplementary Table S1. Number of retrieved gene targets for globospiramine (**1**) and each sensitive cell line from databases.

Compound / Cell Lines	SWISS Target Prediction	PharmMapper	DisGeNET + GeneCards
Globospiramine	100	291	-
MCF-7	-	-	8574
PC-3	-	-	10475
SKOV-3	-	-	1065
KB3.1 or HeLa	-	-	8635

(-) = not applicable. Duplicated genes were counted under SWISS Target Prediction for globospiramine (**1**) targets. DisGeNET and GeneCards genes were combined after duplicate removal.

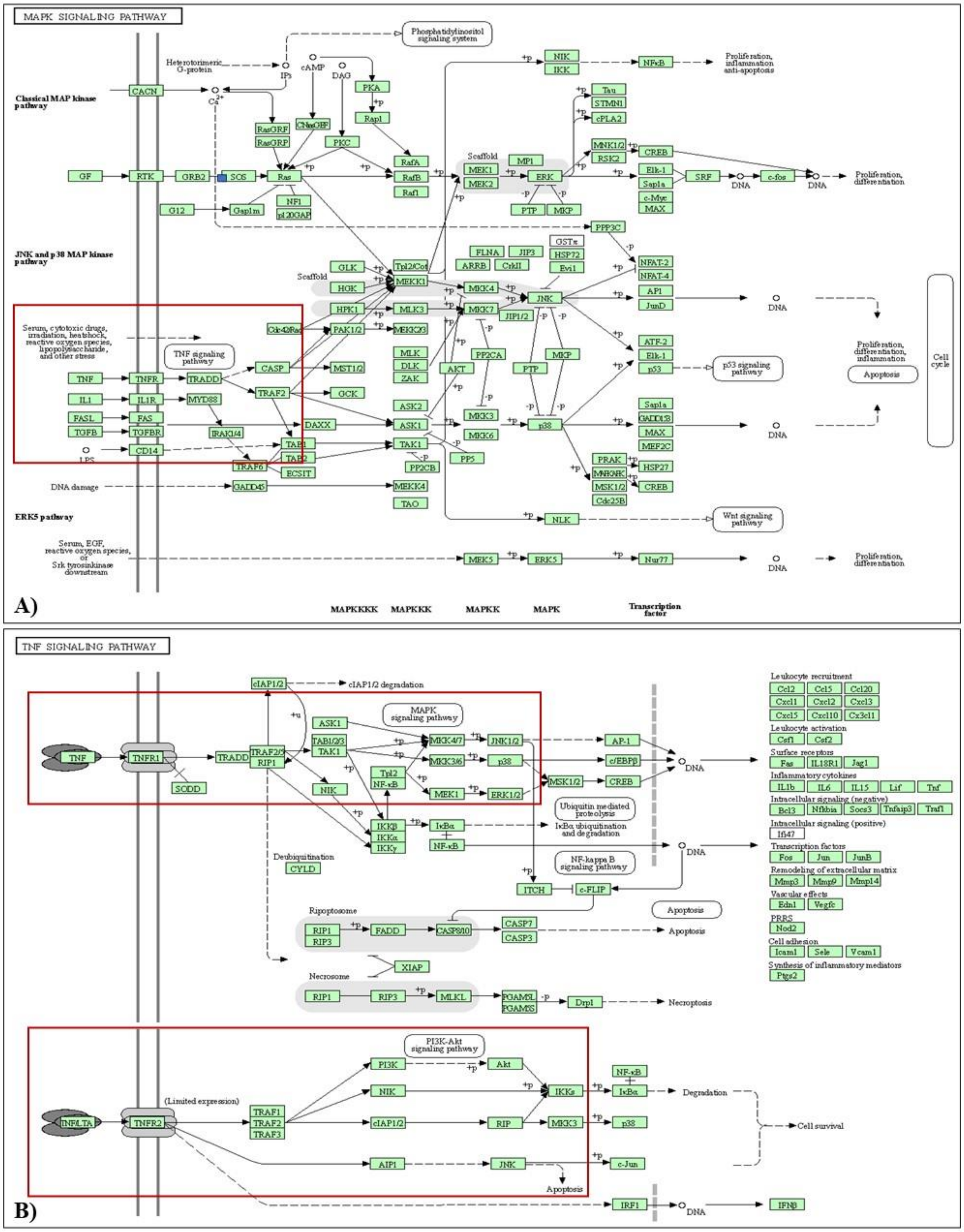

Supplementary Figure S1. Visualized KEGG pathway maps of (**A**) MAPK signaling pathway and (**B**) TNF signaling pathway. MAPK signaling pathway is shown downstream of TNF. Other well-established therapeutic pathway targets are shown in the TNF and PI3K-AKT signaling pathways.
